# Spatial and temporal pattern of changes in the number of GAD65-immunoreactive inhibitory terminals in the rat superficial dorsal horn following peripheral nerve injury

**DOI:** 10.1186/1744-8069-10-57

**Published:** 2014-09-04

**Authors:** Louis-Etienne Lorenzo, Claire Magnussen, Andrea L Bailey, Manon St Louis, Yves De Koninck, Alfredo Ribeiro-da-Silva

**Affiliations:** Department of Pharmacology and Therapeutics, McGill University, 3655 Promenade Sir-William-Osler, Montreal, Quebec H3G 1Y6 Canada; Alan Edwards Centre for Research on Pain, McGill University, Montreal, Quebec H3A 2B2 Canada; Institut Universitaire en Santé Mentale de Québec, Québec, QC G1J 2G3 Canada; Departments of Anatomy and Cell Biology, McGill University, Montreal, Quebec H3A 2B2 Canada

**Keywords:** Pain, Inhibition, Chronic constriction injury, Neuropathic, IB4, GAD65, Non-peptidergic C fibre, Inhibitory interneurons, Synaptic boutons

## Abstract

Inhibitory interneurons are an important component of dorsal horn circuitry where they serve to modulate spinal nociception. There is now considerable evidence indicating that reduced inhibition in the spinal dorsal horn contributes to neuropathic pain. A loss of these inhibitory neurons after nerve injury is one of the mechanisms being proposed to account for reduced inhibition; however, this remains controversial. This is in part because previous studies have focused on global measurements of inhibitory neurons without assessing the number of inhibitory synapses. To address this, we conducted a quantitative analysis of the spatial and temporal changes in the number of inhibitory terminals, as detected by glutamic acid decarboxylase 65 (GAD65) immunoreactivity, in the superficial dorsal horn of the spinal cord following a chronic constriction injury (CCI) to the sciatic nerve in rats. Isolectin B4 (IB4) labelling was used to define the location within the dorsal horn directly affected by the injury to the peripheral nerve. The density of GAD65 inhibitory terminals was reduced in lamina I (LI) and lamina II (LII) of the spinal cord after injury. The loss of GAD65 terminals was greatest in LII with the highest drop occurring around 3–4 weeks and a partial recovery by 56 days. The time course of changes in the number of GAD65 terminals correlated well with both the loss of IB4 labeling and with the altered thresholds to mechanical and thermal stimuli. Our detailed analysis of GAD65+ inhibitory terminals clearly revealed that nerve injury induced a transient loss of GAD65 immunoreactive terminals and suggests a potential involvement for these alterations in the development and amelioration of pain behaviour.

## Introduction

Neuropathic pain is a debilitating condition that arises as a “direct consequence of a lesion or disease affecting the somatosensory system” [1]. Nerve injury is accompanied by many alterations in the dorsal horn of the spinal cord which play a role in driving and maintaining the accompanying pain. One such alteration that remains controversial is the question of whether or not inhibitory interneurons in the dorsal horn are lost in conditions of pain [2, 3]. This is an important question to resolve, as these inhibitory interneurons play an important role in modulating sensory information before it is forwarded to higher brain centers.

Most pain related information is forwarded from the periphery to the central nervous system by unmyelinated primary afferents (C fibres) called nociceptors. They have been divided into two roughly distinct populations based on neurochemical and anatomical criteria: the peptidergic and non-peptidergic C fibres [4–7]. Of particular interest to this study are the non-peptidergic C fibres which bind the isolectin B4 (IB4), express purinergic P_2_X_3_ receptors and possess fluoride-resistant acid phosphatase activity [6]. These non-peptidergic fibres terminate primarily in inner lamina II (LIIi) of the dorsal horn in complex synaptic glomeruli, where they represent the central element and are often postsynaptic to inhibitory interneurons [8–10]. After peripheral injury of the sciatic nerve, IB4-binding is lost and subsequently restored in both the dorsal root ganglia [11] and in the spinal cord [12–15]. The loss of IB4 binding in the dorsal horn corresponds to a bona fide loss of the central terminals of non-peptidergic fibres, as confirmed by electron microscopy (EM) [13], indicating the reliability of this marker.

Inhibitory interneurons represent an important component of dorsal horn circuitry, and form close associations with non-peptidergic fibres. The inhibitory neurotransmitter γ-aminobutyric acid (GABA) is synthesized from glutamate by the enzyme glutamic acid decarboxylase (GAD), and both neurotransmitter and enzyme are found in these inhibitory interneurons. Two distinct isoforms of GAD exist; GAD 67 and GAD 65 and each is encoded for by a different gene, *gad1* and *gad2*, respectively [16]. In the brain, whereas GAD67 is a cytosolic enzyme that is widely distributed throughout the neuron and often labels cell bodies, GAD65 predominantly localizes to the nerve terminal [17, 18]. This differential pattern of staining does not seem to be true in the spinal cord where most axon terminals contain both isoforms [19]. This said, the predominance of each isoform is suggested to vary based on laminar location, with LI and LII containing significantly more inhibitory profiles that largely express GAD65 than deeper laminae, where GAD67 staining is stronger [19]. Recently, GAD65 was shown to be directly implicated in pain mechanisms, as GAD65 knockout mice show impaired synaptic inhibition and sensitized pain behaviour [20].

Neuronal apoptosis in the dorsal horn has been reported in models of neuropathic pain, and it has been suggested that inhibitory interneurons are among the neurons lost [3, 21–24]. As mentioned, this loss of dorsal horn inhibitory neurons remains controversial as others propose that it is primarily microglia [25] and not inhibitory neurons that undergo apoptosis [2, 25, 26]. Regardless of whether or not there is a loss of neurons, remaining inhibitory neurons could either compensate by increasing their number of synaptic profiles or conversely, could shrink by reducing their synaptic connectivity. One way of clarifying the effect of nerve injury on inhibitory neurons in the dorsal horn is to perform a proper quantification of inhibitory terminals. Accordingly, this study was designed to investigate if the number of GAD65-immunoreactive (IR) boutons was altered following a chronic constriction injury (CCI) by means of a polyethylene cuff in rat. IB4 labelling was used as a tool to define the area of the spinal dorsal horn where the afferents affected by the nerve injury terminate and allowed us to quantify the number of GAD65-IR boutons within the area of lesion of IB4 fibres in lamina II (LII) and in adjacent lamina I (LI). Pain-related behaviour was assessed concomitantly with changes in synaptic terminal density. While some studies have examined global changes in GAD expression throughout the spinal cord [3, 22, 27], this is the first to demonstrate changes in the number of GAD65-IR boutons, in the region of loss of nociceptive primary afferents at different time points post nerve injury.

## Results

### Behaviour

**Figure 1:**
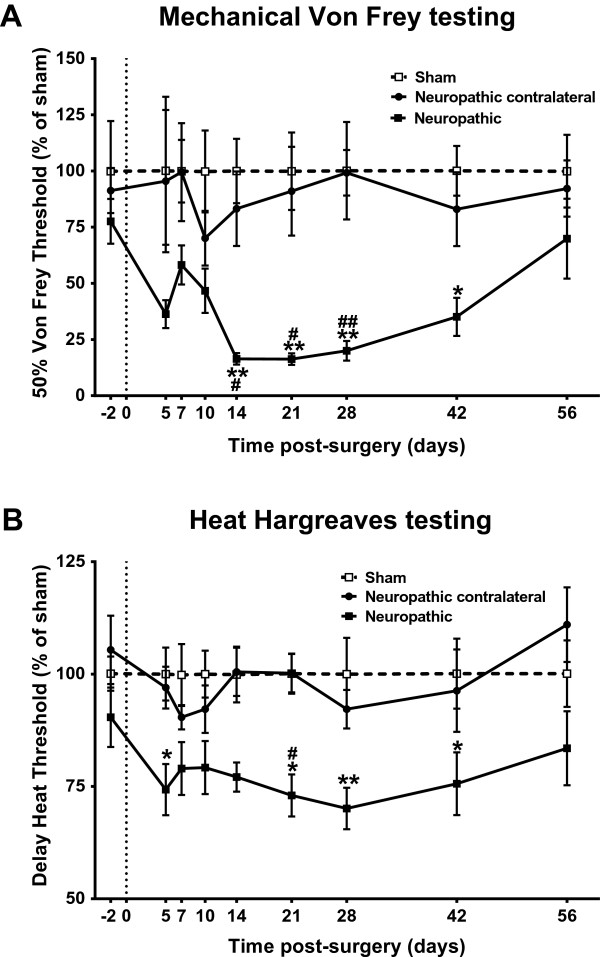
**Behavioural changes following application of the cuff.** Results for the ipsilateral hindpaws are shown for both neuropathic (n = 10) and sham-operated (n = 10) Wistar rats while the contralateral values are shown for neuropathic rats only. Each point represents the mean ± SEM as a percentage of sham values (percentage of sham ipsilateral hindpaw for neuropathic ipsilateral results and percentage of sham contralateral hindpaw for neuropathic contralateral results). **A)** 50% withdrawal threshold to von Frey hairs in neuropathic and sham-operated rats as a percentage of sham values obtained with the up-down method. **B)** Assessment of thermal hyperalgesia using the Hargreaves’ test. Withdrawal threshold to light stimulus as a percentage of sham values. In **A** and **B**, comparison to sham ipsilateral hindpaw *P < 0.05, **P < 0.01 by 2 way ANOVA with Bonferroni correction. Comparison to contralateral hindpaw of cuff animals #P < 0.05, ##P < 0.01.

All of the rats that had undergone cuff surgery exhibited alterations in ipsilateral hind paw posture. Cuff, but not sham animals, held their affected paw in an everted position with the toes plantar-flexed, and avoided bearing weight on it. To facilitate a direct comparison of nociceptive behaviour between sham and cuff animals, all behavioural assessments were normalized to sham controls, with statistics performed on absolute values. Following cuff surgery, animals showed a progressive reduction in paw withdrawal threshold to mechanical stimuli. Test results for mechanical sensitivity are shown in Figure 1A. Ipsilateral to injury, paw withdrawal thresholds (as measured by 50% von Frey withdrawal threshold), decreased starting the 5^th^ day post cuff application, and were significantly different from sham ipsilateral thresholds by 14 days. Mechanical sensitivity reached its maximum 21 days post cuff application when mechanical thresholds were only 16.3 ± 2.6% of sham ipsilateral values. Gradually, thresholds returned to sham levels by 56 days. Cuff contralateral and sham contralateral withdrawal thresholds were not significantly different at any of the time points tested, demonstrating a lack of bilateral hypersensitivity to mechanical stimuli (data not shown).

In addition to changes in mechanical thresholds, animals showed a decrease in withdrawal latencies to a noxious thermal stimulus. Test results for thermal hyperalgesia, measured using the Hargreaves method, are shown in Figure 1B. Starting 5 days after surgery, withdrawal latencies of the ipslateral hindpaw declined steadily, and were significantly different when compared to sham ipsilateral control values at 21 days. Heat hyperalgesia was maximal at 28 days, when thermal thresholds were only 70.1 ± 4.6% of sham ipsilateral values. Like mechanical hyperalgesia, thermal hyperalgesia was still present at day 42 post-surgery and returned only to sham levels by 56 days. Withdrawal latencies decreased slightly contralateral to injury, but were significantly different from cuff ipsilateral values at day 21 post-surgery. Cuff contralateral and sham contralateral withdrawal thresholds were not significantly different at any of the time points tested (data not shown).

### IB4 and GAD labelling in Sham and Cuff Animals

**Figure 2:**
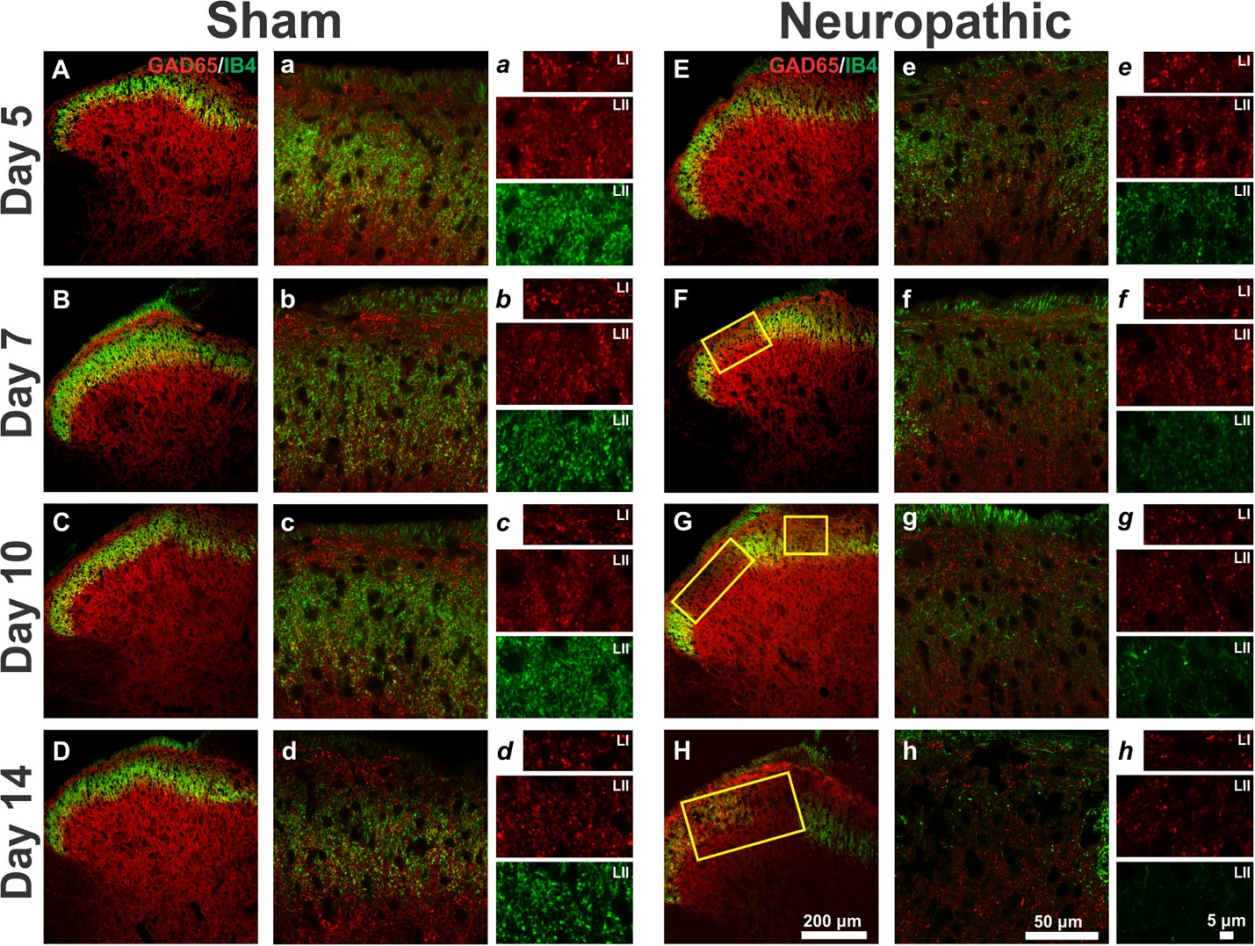
**Representative confocal microscopy images of IB4 and GAD65 immunoreactivity in the dorsal horn of sham (A-D) and neuropathic (E-H) rats ipsilateral to injury at 5, 7, 10 and 14 days after surgery.** Capital letters and lowercase letters denote images taken with 20X and 63X objectives, respectively. Lowercase italic letters show representative images of quantified regions in both lamina I and lamina II, taken with a 63X objective and a zoom factor of 2. IB4-labeling is shown in green and GAD65 in red. Note that for each time point, the image with the largest loss of IB4 staining was chosen for illustration and this IB4 lesion is shown by a yellow rectangle. The smaller regions used for quantification were taken from within these yellow rectangles. In sham animals, IB4 labelling was primarily restricted to a thin band in lamina II, whereas GAD65 labelling was observed throughout the dorsal horn. GAD65-IR boutons were readily observed among the IB4-labelled terminals. Starting at 5 days after surgery **(E)** but more clearly visible from day 7 **(F)** there was a region of depletion of IB4 staining that was not observed in sham animals at any time point **(A-D)**. High magnification images of neuropathic rats show a clear lack of IB4 staining in lamina II from days 7 (**f** and ***f***) to 14 (**h** and ***h***).GAD65-IR boutons are reduced in cuff animals **(** ***e-h*** **)** compared to sham animals **(** ***a-d*** **)**.

**Figure 3:**
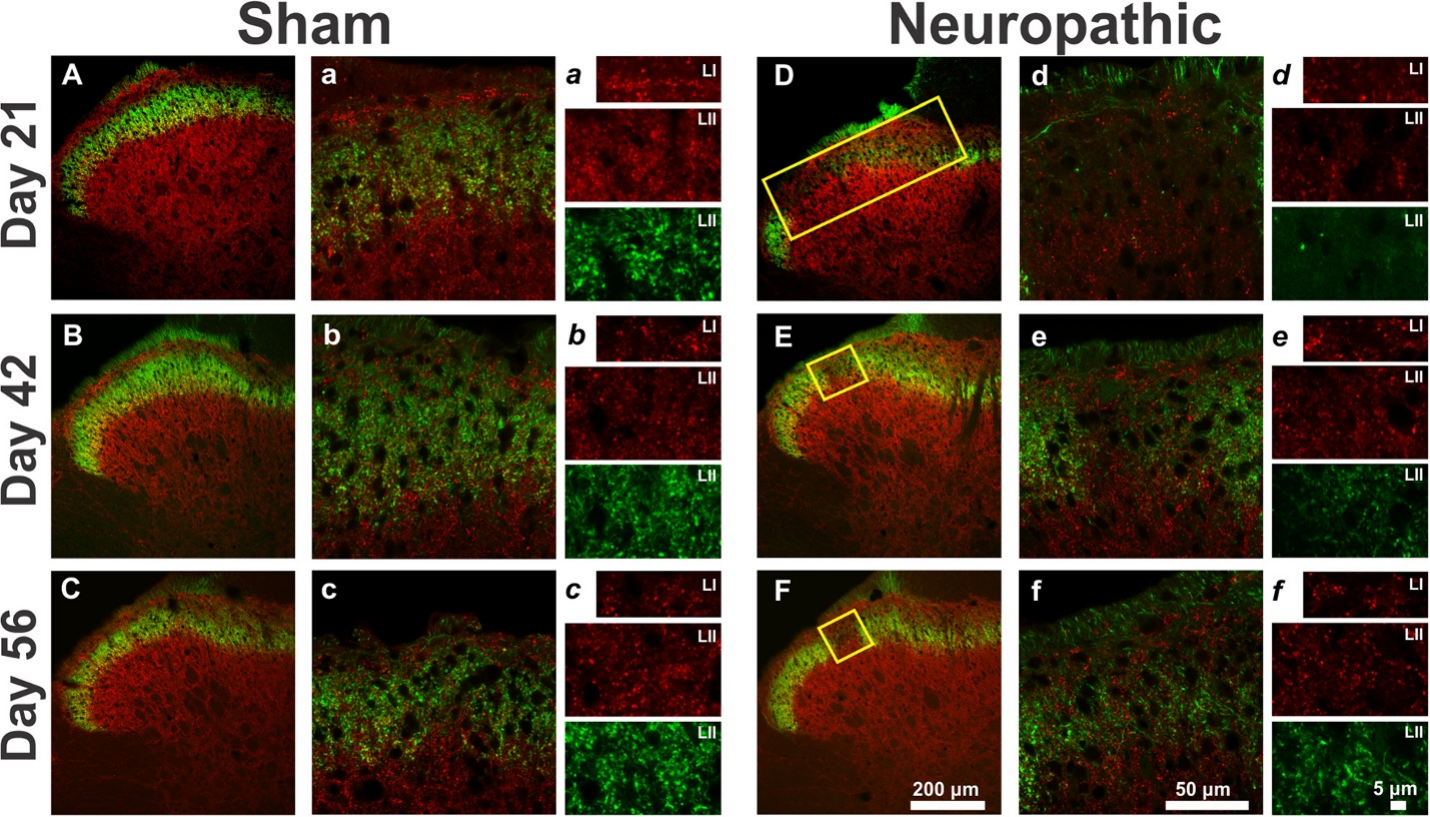
**Representative confocal microscopy images of IB4 staining and GAD65 immunoreactivity from the dorsal horn of sham (A-C) and neuropathic (D-F) rats ipsilateral to injury at 21, 42, 56 days after surgery.** Capital letters and lowercase letters denote images taken with 20X and 63X objectives, respectively. Lowercase italic letters show representative images of quantified regions in both lamina I and lamina II, taken with a 63X objective, and a zoom factor of 2. IB4-labelling is shown in green and GAD65 in red. Note that for each time point, the image with the largest loss of IB4 staining was chosen for illustration and this IB4 lesion is shown by a yellow rectangle. The smaller regions used for quantification were taken from within these yellow rectangles. High magnification images show a clear lack of IB4+ terminals and an important loss of GAD65-IR in lamina II at 21 days (**d** and ***d***) in neuropathic animals. By 42 days (**e** and ***e***) after surgery, IB4-IR and GAD65-IR began to be restored in the lesion, however by 56 days (**f** and ***f***) there were still considerably fewer IB4+ terminals present when compared to sham (**c** and ***c***).

To investigate the changes in GAD65-IR inhibitory terminals in the superficial dorsal horn, a double labelling was performed at various time points after cuff application using a monoclonal antibody directed against GAD65 combined with IB4 conjugated to a fluorochrome. The IB4 labelling was used as a tool to define the location of the lesion that occurs in the dorsal horn after CCI. In this way, the changes in GAD65-IR inhibitory terminals could be quantified in the region where the majority of IB4 fibres terminate, LII, and outside this region, in LI. Morphological studies were performed at 5, 7, 10, 14, 21, 42 and 56 days, allowing us to study the onset of changes, as we had many early time points, and allowed us to discriminate a time at which markers returned to near normal using the 2 later time points. Figure 2 shows representative images of GAD65 and IB4 labelling in sham (low magnification 2A-D, high magnification 2a-d) and cuff animals (low magnification 2E-H, high magnification 2e-h) 5, 7, 10 and 14 days after surgery, while images taken at days 21, 42 and 56 are shown in Figure 3 (sham 3A-C, 3a-c; cuff 3D-F, 3d-f). Panels labelled with lowercase italic letters (Figure 2*a-h* and Figure 3*a-f)* show representative images of the high magnification regions in both lamina I and II that were used for quantification. In these images, GAD65 terminals in red and IB4 terminals in green are most clearly visible.

In sham-operated animals, IB4-binding was mostly restricted to LII, with much less labelling in lamina I. Labelling was particularly intense in LIIi (Figures 2A-D and 3A-C). At greater magnification, IB4 binding was seen to be concentrated in varicosities (Figures 2*a-d* and 3*a-c*). Cell bodies were not labelled. Contrary to IB4 labelling, GAD65 immunoreactivity was observed throughout the entire dorsal horn and its punctate appearance was evident at high magnification (Figures 2*a-d* and 3*a-c*). Although some of the structures we name as varicosities certainly represent GABAergic presynaptic dendrites [9], they cannot be distinguished from GABAergic axonal varicosities by light microscopy. All punctate GAD65-IR structures larger than 0.05μm^2^ were counted, to avoid as much as possible counting cut axons as varicosities or terminals.

**Figure 4:**
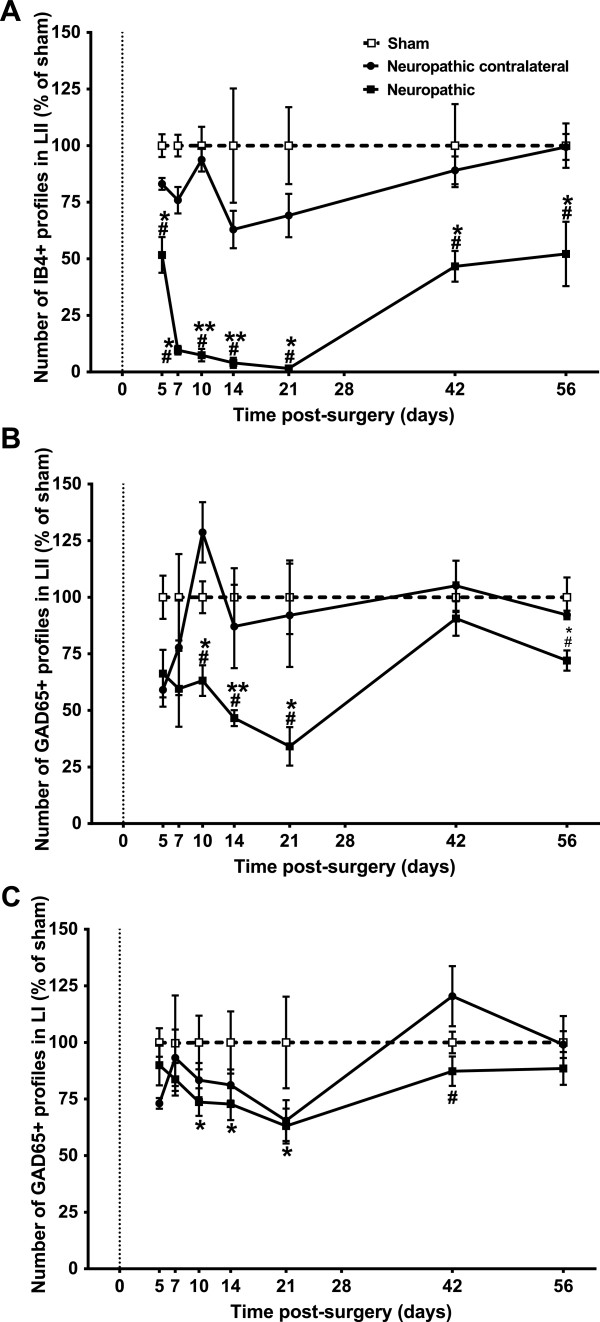
**Quantitative analysis of the number of boutons in the superficial dorsal horn of the spinal cord in sham and neuropathic rats at various times after surgery.** Quantification of terminal density was performed within the region of loss of IB4 staining. The number of ipsilateral profiles is shown for neuropathic and sham rats, with contralateral values shown only for neuropathic animals. Each point represents the mean ± SEM as a percentage of sham values. **A)** Quantitative analysis of the density of IB4-labelled terminals in lamina II in sham and neuropathic rats. Neuropathic rats had a drastic reduction in the number of IB4+ terminals ipsilateral to injury with maximal loss occurring at 21 days followed by a gradual recovery with numbers remaining significantly different from sham at 42, 56 days. There were always significantly fewer IB4+ terminals ipsilateral vs. contralateral to injury. **B)** Quantitative analysis of GAD65-IR terminals in lamina II in sham and neuropathic rats. Neuropathic rats had a progressive reduction in the number of GAD65-IR terminals ipsilateral to injury with significantly fewer profiles than sham at 10 days, and a maximal loss at 21 days. Despite a partial recovery, GAD65-IR terminals remained significantly reduced at 56 days compared to sham, and at 10, 14, 21, and 56 days were significantly lower than contralateral values. **C)** Quantitative analysis of GAD65-IR terminals in lamina I in sham and neuropathic rats. Neuropathic rats had a small but progressive reduction in the number of GAD65-IR terminals ipsilateral to injury with values significantly different from sham at 10, 14 and 21 days and recovery by 42 days. There was a similar loss of GAD65-IR terminals ipsilateral and contralateral to injury. *P < 0.05, **P < 0.01 sham ipsilateral vs. neuropathic ipsilateral #P < 0.05 neuropathic ipsilateral vs. contralateral.

Beginning as soon as 5 days post-surgery, cuff animals exhibited a decrease in IB4-labelled (IB4+) varicosities (compared to sham) ipsilateral to injury. The region of loss of IB4 binding ("lesion" zone, delineated with a yellow rectangle) was most evident in the middle of LII, or more precisely in the intermediate third of the medio-lateral extent of the dorsal horn (Figure 2H), which corresponds to the spinal cord projection of the sciatic nerve [28]. The density of IB4+ terminals in this region of LII was quantified over time in cuff and sham animals and the results are displayed in Figure 4A. Values for the ipsi- and contralateral paws have been normalized to those from sham controls, while statistical analysis was performed using absolute values. Ipsilateral to injury, the density of IB4+ terminals in LII was significantly decreased at all time points studied when compared to sham controls and to the side contralateral to injury. From 5 days, the number of IB4+ profiles progressively declined and reached a minimum at 21 days (Figure 3D), when the IB4+ varicosity density ipsilateral to injury was 1.5 ± 0.3% of sham controls. Gradually over the next 5 weeks, the density of IB4+ terminals increased; however, an area of depletion was still present at 56 days (Figure 3F) when the density of IB4+ terminals reached only 52.1 ± 14.2% of sham controls.

The density of GAD65-IR terminals in LII is displayed in Figure 4B. Cuff application decreased the density of GAD65-IR terminals ipsilateral to injury. While there was a small decrease in the density of GAD65-IR terminals at 5 and 7 days after CCI, it was only at 10 days that the number of GAD65-IR terminals was significantly reduced within the area of IB4+ depletion in LII, when compared with sham controls and to the side contralateral to injury. As seen with IB4, a maximum reduction in GAD65-IR terminals occurred at 21 days, where the density reached 34.1 ± 8.5% of sham controls. While a statistical recovery of GAD65 terminals occurred at 42 days when compared to controls, at 56 days a small, albeit significant reduction in the number of GAD65-IR varicosities was still present as density was 72 ± 4.5% of sham levels.

The density of GAD65-IR terminals was also quantified in LI (Figure 4C), a region which is much less innervated by non-peptidergic fibres [10, 29, 30]. From 5 days, the number of GAD65-IR varicosities progressively declined and reached a minimum at 21 days, when their density ipsilateral to injury was 63.1 ± 7.7% of sham controls. The reduction in GAD65-IR terminals in LI (63.1 ± 7.7%) was much less pronounced than in LII. Contralateral to injury, a similar though much less marked trend was observed, as the density of GAD65-IR terminals in LI was not significantly different between ipsilateral and contralateral sides at days 5, 7, 10, 14, 21, and 56 days.

A non-parametric Spearman correlation was performed to determine if there is a relationship between the loss of IB4+ terminals and the loss of GAD65+ varicosities in LII. To do this, IB4+ bouton mean density was correlated to their corresponding GAD65+ bouton mean density value in every animal. Interestingly, in the density decreasing phase of both entities, the density of IB4+ terminals was significantly correlated with the density of GAD65-IR terminals from 5 to 14 days along the four time points studied (5, 7, 10, 14 day; r = −0.4, p = 0.048, n = 25). As IB4 terminal density decreased, so did GAD65 density; however, this correlation was not observed in the “recovery phase” of the two markers (from day 21 to 56; r = 0.09, p = 0.73, n = 16).

## Discussion

In this study, the time-dependant changes in the number of GAD65-IR inhibitory terminals were examined in the superficial dorsal horn of the rat spinal cord following polyethylene cuff induced CCI of the sciatic nerve. Using specific markers, and restrictive quantification techniques, we show conclusively that cuff application results in a significant but transient loss of GAD65 immunoreactivity, corresponding to inhibitory terminals, within the area of loss of IB4+ boutons in LII and, dorsal to it, in LI. The early time-course of the loss of GAD65-IR terminals correlated well with the loss of IB4+ terminals in LII. Furthermore, the pattern of alterations in terminal densities paralleled the changes in thresholds to both mechanical and thermal stimuli.

It is well known that loss of GABAergic or glycinergic inhibition in the dorsal horn leads to allodynia in naive rats [31], and that loss of inhibitory tone in the dorsal horn might underlie symptoms of neuropathic pain [32]. In light of these results, many studies have explored the mechanisms which might contribute to this altered neurotransmission. Of particular interest was the question of whether or not nerve injury resulted in a loss of inhibitory interneurons. Conflicting evidence quickly emerged. Some studies suggested that GABAergic interneurons were lost after nerve injury as indicated by a loss of GABA immunoreactivity in the dorsal horn [23, 24, 27] and the presence of apoptotic markers [3, 21, 22] while other studies using stereological counts opposed these results by showing that neurons in LI-III were not lost after either CCI [2, 26] or spared nerve injury (SNI) [25]. Independent of a change in the number of GABAergic cells following injury, it is likely that they either retract or extend their distal processes, a phenomenon that has been well described in hippocampal neurons after stress [33].

In this study, GAD65 was used to visualize inhibitory terminals by immunofluorescence and to clarify the effect of nerve injury on inhibitory neurons in the dorsal horn. GAD65 was chosen for a number of reasons. 1. It primarily localizes to nerve terminals [17–19]. 2. In laminae I and II, where this study was focused, inhibitory profiles with high levels of GAD65 are relatively common; this is not the case in more ventral laminae where profiles with high levels of GAD67 dominate [19]. 3. GAD65 knockout mice have impaired GABA synaptic function and a lower baseline pain threshold indicating a direct role for this isoform in both inhibition and pain [20]. Using software parameters that specifically allowed puncta the size of GABAergic boutons with a minimal staining intensity to be counted as inhibitory terminals, we have clearly shown that the density of GAD65+ inhibitory terminals was reduced in the ipsilateral superficial dorsal horn of the spinal cord after cuff injury. The loss of GAD65-IR inhibitory terminals was not constant over time or space. The loss of inhibitory terminals was most significantly reduced at 3–4 weeks and recovered partially by 56 days. Furthermore, the loss of GAD65-IR terminals was greatest in LII, the region where the majority of IB4+ fibres terminate and are lost after nerve injury.

The finding that GAD65-IR density is temporally altered by nerve injury is consistent with a number of complementary findings. Meisner et al. [22] showed that GAD65/67 immunoreactivity in the dorsal horn is reduced at 8 weeks after spinal cord injury (SCI), as are the protein levels of GAD67 and GAD65 at 6 weeks. Moore et al. [3] showed using Western blot that GAD65 protein is transiently reduced in the ipsilateral dorsal horn by CCI and SNI; however, unlike us, they saw a full restoration of GAD65 levels at 4 weeks post CCI. These immunoblotting approaches used only provide a global assessment of GAD protein levels in the entire ipsilateral dorsal horn and likely do not reflect what is happening in LI and LII, regions important in nociceptive transmission. Finally, Eaton et al. [27] show that GAD67-IR cell bodies in LI-III initially decrease in number 3 days post CCI but begin to increase, eventually surpassing levels in intact animals at 8 weeks after nerve injury. The different time-course seen in this study is most likely attributable to the fact that cell bodies, rather than terminals, were counted. The major advantage of our study over the previous studies is that we were able to specifically quantify the number of inhibitory terminals at various time points, within the specific area in the spinal dorsal horn where the afferents affected by the nerve injury terminate.

It is possible that the loss of GAD65 immunoreactivity does not necessarily reflect a loss of inhibitory terminals, but instead simply a down regulation of the protein. Because it has been shown that some boutons in the superficial dorsal horn express high levels of GAD65 and low levels of GAD67 [19], it is possible that these boutons would be unable to synthesize GABA after GAD65 downregulation, effectively rendering these synapses non-functional (as far as GABAergic transmission is concerned). One limitation of the current study is that we investigate the changes in GAD65 immunoreactivity only, and do not consider GAD67. While some studies suggest that GAD65 is more dramatically reduced [3], others report that GAD65 and 67 are similarly decreased by nerve injury [22]. If GAD67 is unchanged or even up regulated, some inhibitory synapses could remain functional. Contrary to this, the frequency of inhibitory postsynaptic currents (mIPSCs) is reduced by CCI [3] and in GAD65 knockout animals [20], indicating that inhibitory neurons release less GABA in these models. To more globally assess the entire GABAergic inhibitory terminal population in neuropathic pain, antibodies against GAD67 could be used in combination with those against GAD65 or even by using antibodies that indiscriminately recognize both isoforms. Alternatively, the vesicular GABA transporter (VGAT), which acts as an anatomical marker for inhibitory terminals [34, 35], could be used.

One of the most interesting and relevant findings of this study is that the behavioural results matched well with the loss and recovery of both IB4+ and GAD65-IR terminals. As the density of both IB4+ fibre terminals and GAD65-IR inhibitory terminals decreased from 5–21 days, sensitivity to both mechanical and thermal stimuli increased. As GABAergic inhibitory terminals are known to be presynaptic to the central boutons of type Ia glomeruli [9, 36], it is hypothesized that the GAD65-IR terminals might retract and become lost after glomerular IB4+ central terminals undergo degenerative atrophy. This is supported by the fact that we found that loss of GAD65-IR terminals was significantly correlated with the loss of IB4+ terminals in LII from 5–14 days after cuff placement. Between 14 and 28 days, a time at which both IB4+ and GAD65-IR terminals were maximally depleted, hypersensitivities were greatest. It is possible that the disrupted synaptic organization in the dorsal horn at this time might drive the development of neuropathic pain. In normal animals, nociceptive information is forwarded to the spinal cord via two distinct populations of C fibres: the peptidergic and non-peptidergic. Evidence suggests that nerve injury produces more profound and longer lasting changes to both the central and peripheral terminals of non-peptidergic C fibres in comparison to the peptidergic population, which appears to be more resilient [13, 37–39]. We suggest that nociceptive information following nerve injury might be preferentially transmitted through the less affected peptidergic population. Given that peptidergic afferents do not receive axo- or dendroaxonic synapses from GABAergic neurons and therefore undergo less direct inhibition [8], it is conceivable that nociceptive signals are more easily transmitted from these remaining peptidergic fibres to projection neurons. It might be argued as well that as the loss of IB4+ terminals and of GAD65-IR is parallel, the loss of inhibition would be on terminals that disappeared with the lesion. However, such reasoning would assume that the major synaptic targets of GAD65-IR boutons in LIIi are the central boutons of Type Ia synaptic glomeruli, which is far from the truth. Indeed, it should be kept in mind that the great majority of GABA- and glycine-containing terminals are presynaptic to spinal neurons, not to primary afferent terminals [36, 40].

In LI, despite the few IB4 fibres that terminate there [30], the loss and recovery of GAD65 terminals followed the same time course as within LII, albeit less severe. These data from LI suggest that it is mostly the inhibitory terminals that contact dorsal horn neurons that are lost after CCI. In this way, the loss of inhibitory terminals could result in impaired dorsal horn inhibition and altered nociception.

Finally, we suggest that the amelioration in behaviour seen at later time points might be in part due to a recovery of the normal synaptic architecture of type Ia glomeruli and the reestablishment of an equilibrium between the peptidergic and non-peptidergic populations. In line with this idea, regenerative proliferation of IB4+ terminals is observed after cuff [13], and 40 days after crush injury growth cones of primary afferent terminals have been shown to re-establish synapses with dendritic processes from substantia gelatinosa neurons [41]. Future studies using EM would be required to determine if GAD65-IR terminals resume proper connectivity and presynaptic inhibition to non-peptidergic C fibres. Recently it was shown that when immature GABAergic interneurons were transplanted and integrated into spinal cord circuitry, the mechanical hypersensitivity associated with SNI was reversed [42]. These results suggest a potential therapeutic role for restoring normal inhibitory function in the dorsal horn in chronic pain conditions.

## Conclusions

In this study we show conclusively that GAD65 immunoreactivity is transiently lost, suggesting a loss of inhibitory terminals, in the superficial dorsal horn of the rat spinal cord following polyethylene cuff-induced lesion of the sciatic nerve. The reduction of inhibitory terminals was most severe in LII and the time course correlated with both the loss of IB4+ terminals and with the altered thresholds to both mechanical and thermal stimuli. Further studies are required to assess whether or not GABAergic terminals return to their correct location, and re-establish a proper inhibitory synaptic connectivity with IB4+ central terminals. Additionally, future studies should quantify the proportion of lost inhibitory terminals that were in contact with IB4+ terminals versus dorsal horn neurons.

## Methods

In total, 88 male Wistar rats (200-300 g; Charles River Canada) were used in this study. Animals were maintained on a 12:12 hour light: dark cycle and received food and water ad libitum. Experiments were performed during the light cycle. All protocols were approved by the McGill University Animal Care Committee and complied with the guidelines set by the Canadian Council on Animal Care and International Association for the Study of Pain.

### Induction of neuropathic pain

Neuropathic pain was induced using a variation of the Mosconi and Kruger [43] technique. In this model, a unilateral chronic constriction of the sciatic nerve is achieved by placing a single polyethylene cuff of fixed diameter around the nerve [44]. All surgery was done under aseptic conditions. Animals were anaesthetized under 5% isoflurane in O_2_ in an induction chamber, and maintained at 2.5% isoflurane throughout the remainder of the surgery. The animals were shaved on their left side below the pelvis. The left common sciatic nerve was exposed via blunt dissection through the biceps femoris muscle. The sciatic nerve was isolated from surrounding fascia using a glass probe, and a 4-6 mm section of the nerve was elevated to allow for placement of the cuff around the nerve. The cuff consisted of a 2 mm piece of split PE-60 polyethylene tubing with an inner diameter of 0.76 mm (Intramedic PE-60, Intramedic, Fisher Scientific, Canada). The muscle and shaved skin layers were closed separately using 4–0 Vicryl absorbable suture (Ethicon, Johnson & Johnson, New Jersey, USA) and an antibiotic ointment was applied. Sham operated rats served as controls, and underwent the same procedure but did not receive any nerve manipulation. Animals were returned to their home cages and were allowed to recover for 5 days before undergoing behavioural testing.

### Behavioural testing

Behavioural testing was performed on 20 Wistar rats, 10 cuff and 10 sham animals. Each animal was followed throughout the following time points: 5, 7, 10, 14, 21, 28, 42 and 56 post surgery.

#### Mechanical Allodynia

Using the up-down method, responses to mechanical stimuli were tested with von Frey filaments of increasing stiffness [45]. Rats were placed in a transparent cage atop a mesh floor and were allowed to acclimatize for 2 days prior to baseline testing for 15 minutes each day. Baseline thresholds were taken 2 days prior to cuff surgery. Von Frey filaments (0.6, 1, 1.4, 2, 4, 6, 8, 10, 15, 26 g) were applied serially in ascending order of strength to the plantar surface of the hindpaw with enough force to elicit a slight bend in the filament. Each filament was applied for 5 seconds or until a flexion reflex occurred. An acute withdrawal of the paw was considered a positive response, and signalled the application of the next weaker filament. In the absence of a paw withdrawal response, termed a negative response, the next stronger stimulus was presented. The resulting pattern of positive and negative responses was tabulated, where X = withdrawal and 0 = no withdrawal. The 50% paw withdrawal threshold (grams) was calculated as (10^[*X*^_f_^+ *kδ*]^)/10000 where *X*_f_ = value (in log units) of the final von Frey hair used, *k* = value for the pattern of positive/negative responses and *δ* = mean difference in log unit between stimuli (here, *δ* = 0.224).

#### Heat Hyperalgesia

The Hargreaves test, which measures the latency of paw withdrawal from a radiant heat source, was used to assess thermal hyperalgesia [46]. Animals were placed in a transparent plastic cage on top of a glass plate. For 2 days prior to baseline testing, animals were habituated to the apparatus for 15 min each day. On baseline test day, which occurred 2 days prior to cuff surgery, the plantar surface of the hindpaw was exposed to a beam of radiant heat (adjusted to 30% of its maximum) applied through the glass floor. The light beam is controlled by a photo-cell switch which automatically turns off the light when the rat lifts its limb. This apparatus allows for the measurement of paw withdrawal latency. To prevent tissue damage, a cut-off of 20.48 seconds was used. Testing was performed on both ipsi- and contralateral paws, and measurements reflect the average of 3 trials, conducted at 5 minute intervals.

### Tissue preparation

A separate group of animals was used for immunohistochemistry. At 5, 7, 10, 14, 21, 42 and 56 days post-surgery, rats were deeply anaesthetized with Equithesin and transcardially perfused with 4% paraformadehyde (PFA) in 0.1 M phosphate buffer (PB), pH 7.4, for 30 min. For each time point, 3–5 sham and 4–8 cuff animals were used. The spinal cords were removed and post-fixed in 4% PFA for 2 hours and cryoprotected in 30% sucrose in 0.1 M PB overnight. The following day, 35 μm transverse sections from the 4^th^ and 5^th^ lumbar segments (L4 and L5) were cut on a freezing sledge microtome (SM2000R, Leica Microsystems, Ontario, Canada) and collected in phosphate-buffered saline (PBS) containing 0.2% Triton X-100 (PBS-T).

### Double labelling

Double labelling immunofluorescence was performed to explore the changes in GAD65 immunoreactivity both within and outside of the zone of the IB4 lesion that occurs following CCI. Non-specific staining was blocked by pre-treatment for 60 minutes with 10% Normal Donkey Serum (NDS) (Jackson ImmunoResearch, Pennsylvania, USA) diluted in PBS-T. Sections were then treated with a mixture of mouse monoclonal anti-GAD-65 antibody (1:1000, Millipore/Chemicon, Catalog No. MAB351, Lot No. 0510013666) and IB4 conjugated to Alexa Fluor 488 (1:200, Invitrogen/Molecular Probes; Catalog No. I21411, Lot No. 38753A) in 5% NDS in PBS-T, and allowed to incubate overnight at 4°C. On day 2, donkey anti-mouse IgG antibody conjugated to Rhodamine Red X (1:200, Jackson ImmunoResearch, Catalog No. 715-296-150, Lot No. 70290, 73632) was pre-absorbed with 10 mg/ml of acetone rat brain powder (Sigma, USA) in 5% NDS for 30 min at 37°C. This preparation was vortexed every 10 min, followed by centrifugation at 4000 r.p.m for 20 minutes at 4°C. Following washing, sections were incubated for 2 hours in this mixture. Sections were washed in 0.01 M PBS, mounted on gelatin-subbed slides, and coverslipped using Aquapolymount (Polysciences, Pennsylvania, USA).

### Quantification of IB4+ non-peptidergic and GAD65+ inhibitory terminals by image analysis

Sections from sham and cuff animals were examined using a Zeiss LSM 510 confocal scanning laser microscope (Zeiss Canada) equipped with argon and helium-neon lasers. Appropriate filters were selected for the separate detection of Alexa 488 and Rhodamine Red X using a multi-track scanning method. Twelve-bit images were taken with either a 20X water-immersion or a 63X oil-immersion objective lens. To ensure consistency among samples, all parameters of laser power, pinhole size and image detection were kept unchanged. The chosen parameters were set so that the detection of the staining was maximal while avoiding pixel saturation. For quantification, the channels corresponding to each staining were exported separately as TIFF files. The images were then analyzed with an MCID Elite Image analysis system version 7 (Imaging Research Inc., Ontario, Canada).

Quantitative analysis was performed on 6 tissue sections from each animal. Analysis was performed within the region of loss of IB4 staining which occurs predominantly in LII in the intermediate third of the medio-lateral extent of the dorsal horn. This IB4 lesion is denoted by a yellow box in Figures 2 and 3. To analyze the density of IB4- and GAD65-labelled terminals in the superficial dorsal horn (LI and LII), a rectangle measuring 15 X 40 μm was placed with the longer side at the edge of the white matter for LI, and a rectangle measuring 30 X 50 μm was placed horizontally at 50 μm from the white matter within the region of decreased IB4 staining for each animal. On the contralateral side, these rectangles were placed in a position that mirrored their location on the ipsilateral side. In sham animals, where no IB4 lesion is present, these rectangles were placed where the IB4 lesion normally occurs in neuropathic animals. The number of IR profiles per unit area was calculated by the MCID software. To facilitate the detection of the labelled boutons and to differentiate them from background, we used a function in the software named “target accentuate”, which diminishes the overall contrast of the image but decreases the contrast of smaller structures (e.g. boutons) to a lesser extent than larger structures. In order to compensate for failing to count overlapped varicosities, the software performed a correction based on the average area of each type of varicosity (for IB4 = 0.3 μm^2^, for GAD65 bouton = 0.2 μm^2^), to obtain an estimated varicosity count. Overlapping boutons were not frequent with the reduced optical section thickness used. Furthermore, there was no significant difference in average bouton size from sham and lesioned animals. Most importantly, the software was set to eliminate punctae with areas below 0.05 μm^2^ to ensure that cut axons were not counted as axonal varicosities. We have used this approach in other publications see e.g. [47].

### Statistical analysis

All values are expressed as a percentage of sham controls; however, statistical analysis was performed using absolute values. Ipsilateral hindpaw withdrawal thresholds of sham and cuff animals were analyzed using a 2 way ANOVA with a Bonferroni post-hoc test. For immunohistochemistry, the numbers of profiles were statistically analyzed using a one way ANOVA t-test to compare sham to cuff animal ipsilateral values. A paired one way ANOVA t-test was used to compare ipsilateral and contralateral values in each cuff animal. To investigate a correlation between IB4 and GAD65 densities, a non-parametric Spearman correlation was performed. Significance was set at p < 0.05.

### Antibody specificity and controls

In all immunostainings, omission of primary antibodies resulted in negative stainings. The anti-GAD65 monoclonal antibody recognizes the 65 kDa isoform of GAD, and was generated by immunizing mice with purified GAD from rat brain. The mouse anti-GAD65 monoclonal antibody has been well characterized by Chang and Gottileb where it was shown to specifically recognize rat GAD65 on Western blots [48]. Atkinson et al. showed that this monoclonal antibody stained terminals in the rat central nervous system [49].
